# Epidemiology and management of hepatitis B and C in primary care in the Netherlands: data from the Rijnmond Primary Care database

**DOI:** 10.1093/fampra/cmac070

**Published:** 2022-07-23

**Authors:** Sylvia M Brakenhoff, Robert A de Man, Robert J de Knegt, Patrick J E Bindels, Evelien I T de Schepper

**Affiliations:** Department of Gastroenterology and Hepatology, Erasmus MC, University Medical Center, Rotterdam, The Netherlands; Department of Gastroenterology and Hepatology, Erasmus MC, University Medical Center, Rotterdam, The Netherlands; Department of Gastroenterology and Hepatology, Erasmus MC, University Medical Center, Rotterdam, The Netherlands; Department of General Practice, Erasmus MC, University Medical Center, Rotterdam, The Netherlands; Department of General Practice, Erasmus MC, University Medical Center, Rotterdam, The Netherlands

**Keywords:** elimination, general practice, guideline, incidence, prevalence, viral hepatitis

## Abstract

**Background:**

The Dutch guideline for general practitioners (GPs) advises biannual surveillance of hepatitis B (HBV) patients and referral of every hepatitis C (HCV) patient. We aimed to study the prevalence, incidence, and the management of hepatitis B and C in primary care.

**Methods:**

This is a retrospective cohort study using the Rijnmond Primary Care database (RPCD), including health care data of medical records of GPs of approximately 200,000 patients in the area of Rotterdam, the Netherlands. Patient records were selected based on laboratory results, International Classification of Primary Care (ICPC) codes, and free-text words.

**Results:**

In total, 977 patients were included: 717 HBV, 252 HCV, and 8 HBV/HCV coinfected patients. Between 2013 and 2019, the prevalence of HBV and HCV declined from 5.21 to 2.99/1,000 person-years (PYs) and 1.50 to 0.70/1,000 PYs, respectively. We observed that the majority of the patients had been referred to a medical specialist at least once (71% HBV and 89% HCV patients). However, among chronic patients, we observed that 36.2% of the HBV patients did not receive adequate surveillance by their GP (≥2 alanine aminotransferase checks within 3 years) or a medical specialist. In addition, 44.4% of the HCV patients had no record about successful antiviral treatment.

**Conclusions:**

This study demonstrated a declining prevalence in viral hepatitis B and C in primary care in the Netherlands. However, a substantial part of the patients did not receive adequate surveillance or antiviral therapy. It is therefore crucial to involve GPs in case finding and in follow-up after treatment.

**Key messages**

- Viral hepatitis B and C are associated with cirrhosis and liver cancer.
- General practitioners (GPs) play a key role in case finding and surveillance.
- In the last couple of years, the treatment options are significantly improved.
- Therefore, the WHO adopted a strategy aimed to eliminate viral hepatitis.
- This is a study using a large health care database of medical records of GPs.
- We observed that the prevalence and incidence of hepatitis B/C is declining.
- However, many patients did not received adequate surveillance or therapy.
- Therefore, GPs must be involved in case finding and follow-up after treatment.

## Introduction

Chronic infection with viral hepatitis B or C is a global health threat, as it is associated with the development of liver cirrhosis and primary liver cancer (hepatocellular carcinoma) (1,2). Around the world, the prevalence of an infection with hepatitis B virus (HBV) or hepatitis C virus (HCV) is estimated at, respectively, 296 million and 58 million (1,2). In the Netherlands, the prevalence is estimated at 0.3% (40,000 individuals) for HBV and 0.2% (28,000 individuals) for HCV in 2016 (3,4).

During the last years, the treatment of viral hepatitis B and C has improved significantly. Suppression of the hepatitis B virus can be achieved with nucleos(t)ide analogs (NAs) (5,6) and eradication of the hepatitis C virus with direct-acting antiviral agents (DAAs) (7–10). Viral suppression or eradication halts further progression of the liver disease and improves life expectancy (11,12). Therefore, the World Health Organization (WHO) has adopted a Global Health Sector Strategy on viral hepatitis in 2016, aimed to eliminate viral hepatitis B and C as a public health threat by 2030 (13). In the Netherlands, these targets have been implemented in a National Hepatitis Plan (14). Despite these effective treatment options and harm reduction strategies in the last decade(s), the annual mortality does not change and approximately 500 individuals die in the Netherland yearly (3). In Europe, the incidence and mortality rates differs considerably between different countries. The highest prevalence is observed in countries in eastern and southern Europe, especially among high-risk groups such as people who inject drugs (PWID) and men who have sex with men (MSM) (15). In 2015, the annual mortality was 1.3 per 100,000 in Europe. The highest mortality rates were observed in Italy, Germany, and Spain (accounted for two-thirds of all chronic hepatitis related deaths in Europe) (16). Thus, adequate surveillance and treatment of patients with viral hepatitis is important.

General practitioners (GPs) play a key role in case detection and management of viral hepatitis. Indication for HCV and HBV screening include migrants originating from high endemic countries, PWID and MSM, as well as patients with elevated liver enzymes. Patients with an active infection should be referred to a medical specialist to evaluate the presence of liver-related complications and initiate antiviral therapy when indicated. However, several studies demonstrated that many patients who are at risk for viral hepatitis infection are not screened accordingly, resulting in many undiagnosed (17,18).

This could be explained by the fact that most clinical practice guidelines in European countries lack information about the management of patients with viral hepatitis in primary care. In a semiquantitative study, a few GPs in Germany, Spain, and Italy have indicated to be involved in monitoring of serum liver enzymes and refer hepatitis B patients based on clinical indicators (such as hepatitis B e antigen [HBeAg], alanine aminotransferase [ALT], HBV DNA, and comorbidities) (19,20). However, this study also highlighted nonuniform practices in screening and monitoring of patients with viral hepatitis (20). In 2016, the Dutch guideline for viral hepatitis of the Dutch College of General Practitioners has been updated (21). Whereas the outdated guideline recommended HBV surveillance for at least 3 years, which could be ceased if no sign of hepatitis (ALT elevation) or HBeAg levels were negative, the updated guideline recommends lifelong surveillance (including ALT measurement every 6 months and hepatitis B surface antigen [HBsAg] measurement every 3 years) and referral of patients with an active viral hepatitis B or C to an hepatitis treatment center (21). However, the compliance with the Dutch guideline, as well as the prevalence of viral hepatitis B and C in primary care in the Netherlands, is unknown.

In this study, we therefore aim to provide insight in the prevalence of viral hepatitis B and C in a multiethnic area in the Netherlands, as well as in the management of hepatitis B and C patients in primary care.

## Methods

### Study design

This is a retrospective cohort study using the Rijnmond Primary Care database (RPCD). The RPCD is a region specific product of the Integrated Primary Care Information (IPCI) database, supervised by the department of General Practice of the Erasmus MC, University Medical Center, Rotterdam, the Netherlands. More information about the IPCI database has been prescribed in detail elsewhere (22). This is a longitudinal observational dynamic database containing medical records of over 200,000 patients from the area of Rotterdam, the Netherlands. These pseudonymized medical records contain demographics, medical notes (free text), diagnoses (including International Classification of Primary Care [ICPC] codes), laboratory results, and drug prescriptions that are routinely collected by GPs. The database included approximately 25% of the population of the area of Rotterdam, equally distributed across the region and including neighborhoods with different socioeconomic and migration levels. Rotterdam is a dense urban, multiethnic area; 52% of the residents have a non-Dutch background and the nearest GP practice has an average distance of 0.6 km (0.37 miles) (23,24). The study period started on 2013 Dec 1 and ended on 2019 Dec 31.

### Study population

Patient records were selected based on laboratory results, ICPC codes and/or key words for hepatitis B and C. The ICPC classification is managed by the Dutch College of GPs and adopted by all Dutch GPs (25). The database covers laboratory results ordered by the GP and is not linked to hospital records. For hepatitis B, laboratory results included a positive result of HBsAg, ICPC codes D72.02 (acute hepatitis B) or D72.04 (chronic hepatitis B). For hepatitis C, laboratory results included a positive result of HCV antibodies (anti-HCV), ICPC codes D72.03 (acute hepatitis C) or D72.05 (chronic hepatitis C). Patients were excluded if they were identified by an HBV ICPC code, but were also (i) vaccinated for HBV (ATC code J07BC01 or J07BC20), (ii) had a negative HBsAg results within 24 weeks after the ICPC code registration date, or (iii) based on free-text words (including words for prior hepatitis B, vaccination HBV).

After selecting the patients that met these inclusion criteria, the medical charts were reviewed. Cases were labeled as certain cases or uncertain cases (for example if a patient was identified by an ICPC code, but without any additional information in the medical file regarding medical notes and/or laboratory results). All cases were manually categorized as viral hepatitis B (stratified as acute hepatitis B, chronic hepatitis B, and hepatitis B reactivation), hepatitis C (stratified as acute hepatitis C and chronic hepatitis C) and chronic HBV/HCV coinfection. Acute hepatitis B/C was defined as HBsAg positivity for hepatitis B and HCV RNA positivity (or anti-HCV in case of absent HCV RNA testing) for hepatitis C, typically with concomitant jaundice and/or elevation of serum liver enzymes, that occurred within 6 months after viral exposure. Chronic hepatitis B/C was defined as serum HBsAg/HCV RNA positivity (or anti-HCV in case of absent HCV RNA testing) of at least 6 months. HBV reactivation was defined as HBsAg positivity and elevated HBV DNA, in previously HBsAg negative patients but anti-HBc positive patients who underwent high-risk-immunosuppressive treatment. If the medical diagnosis was clearly formulated in a GP note or letter from a hepatitis specialist, this diagnosis was adopted as well. Date of first diagnosis was extracted from the RPCD, but manually altered during chart review if it was evident that the date of diagnosis was different.

### End points

First, we studied the prevalence and incidence of viral hepatitis B and C in the study period. Follow-up ended when a patient transferred out of the GP practice, died, or when the end of the study period was reached, whichever occurred first. In addition, follow-up ended as well when a patient cleared the virus spontaneously or after antiviral treatment, i.e. HCV RNA or HBsAg negativity in previous HCV RNA or HBsAg positive patients.

Next, the management of viral hepatitis B and C patients was studied. For chronic hepatitis B patients, management was categorized as surveillance by medical specialist (at least one letter or note of medical specialist about viral hepatitis B), surveillance by GP (at least 2 ALT checks within 3 years), and no surveillance. The management was only determined in a subcohort of certain cases with a chronic infection. For hepatitis C, the number of referrals and antiviral treatment was studied. Prescriptions for antivirals were extracted using the ATC codes: J05AP (antivirals for treatment of HCV infections; direct-acting antiviral agents), J05AF (nucleoside and nucleotide reverse-transcriptase inhibitors), and L03AB (interferons). Subsequent curation rate (HBV suppression with or without antivirals, HBsAg loss among patients with hepatitis B, and sustained virological response [SVR] or spontaneous clearance for hepatitis C patients), with the corresponding date, was recorded.

### Statistical analysis

Descriptive data were described as numbers (with percentages), medians (IQR), and means (±SD). Analyses were performed in the overall included study population, as well as a subpopulation of certain cases. Incidence was calculated by dividing the number of incident cases by the midterm population at risk (person-years [PYs] at risk within the study population on July first) (26). Prevalence was calculated as year-prevalence proportion, using the number of patients with diagnosis of hepatitis B or C divided by the total number of PY. Both certain and uncertain patients were included for the prevalence/incidence calculation. IBM SPSS for Windows version 27.0 (SPSS Inc., Chicago, IL, USA) was used for statistical analysis. Graph Pad Prism version 5 for Windows (GraphPad Software, San Diego, CA, USA) was used for graphical representation of the results.

## Results

### Study population

**Fig. 1.**
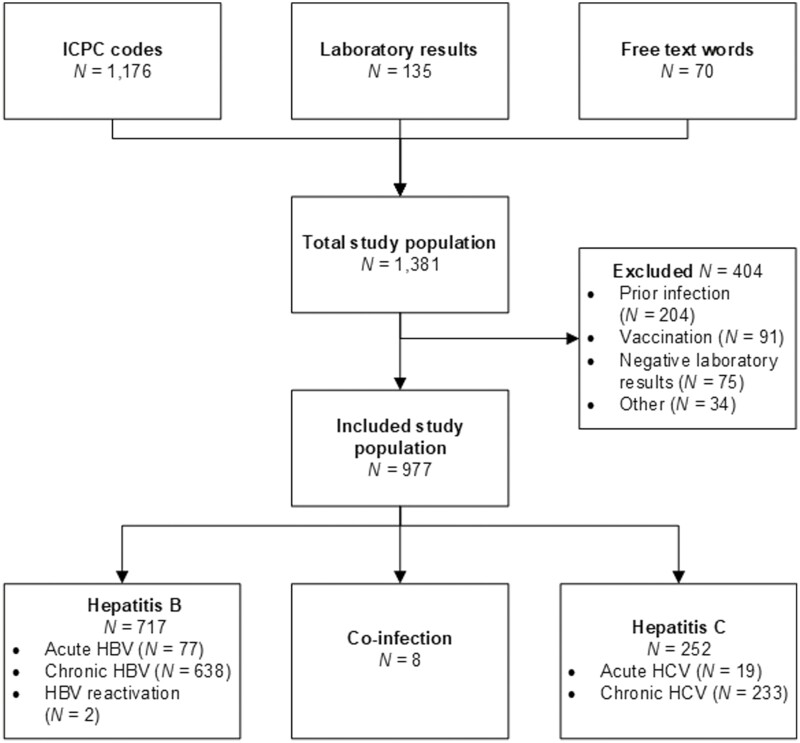
Flowchart of the included study population.

In total, 1,381 patients were identified by the initial search. After reviewing the medical records, 977 patients were included: 717 HBV, 252 HCV, and 8 HBV/HCV coinfection (Figure 1). Baseline patient characteristics are displayed in Table 1. The mean follow-up period was 55 months (IQR 19-98; Supplementary Table 1). After manual validation of these 977 patients, 809 were classified as certain cases: 588 HBV, 214 HCV, and 7 HBV/HCV coinfection.

**Table 1.** Patient characteristics (all patients, *N* = 977)

	Hepatitis B *N* = 717	Hepatitis C *N* = 252	Coinfection *N* = 8
Sex (male), *N* (%)	384 (53.6)	170 (67.5)	7 (87.5)
Age at diagnosis, median (IQR)	37 (27–47)	47 (40–53)	36 (33–45)
Body mass index (kg/m^2^), mean ± SD	27.0 (±5.4)	26.6 (±5.4)	24.5 (±5.0)
Diagnosed by, *N* (%)			
Known infection	251 (35.0)	86 (34.1)	3 (37.5)
Primary care	367 (51.1)	113 (44.8)	4 (50.0)
Hospital	55 (7.7)	40 (15.9)	1 (12.5)
Obstetrics	24 (3.3)	1 (0.4)	0 (0)
Other	20 (2.8)	12 (4.8)	0 (0)
Liver-related comorbidities, *N* (%)			
Compensated cirrhosis	17 (2.4)	27 (10.7)	0 (0)
Decompensated cirrhosis^a^	11 (1.5)	16 (6.3)	0 (0)
Liver transplantation	1 (0.1)	1 (0.4)	1 (12.5)
Hepatocellular carcinoma	14 (2.0)	4 (1.6)	1 (12.5)

Decompensated cirrhosis was defined as ascites, esophageal or fundus varices, jaundice, and/or hepatic encephalopathy.

### Incidence and prevalence of hepatitis B and C

**Fig. 2.**
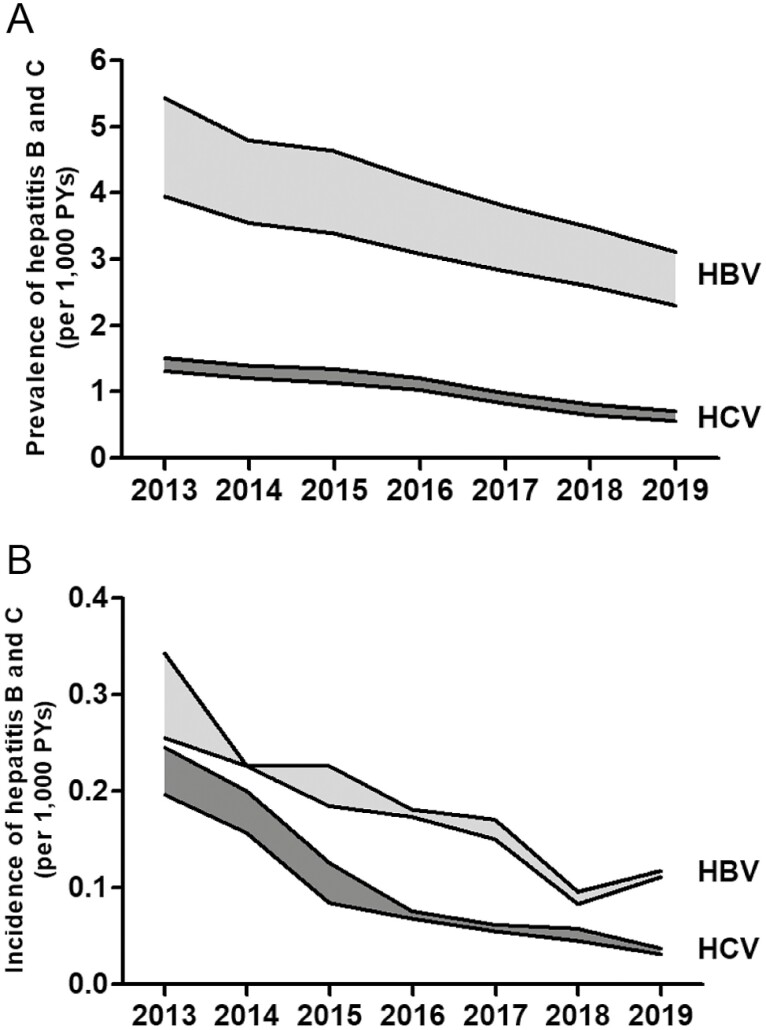
Prevalence (A) and incidence (B) of hepatitis B and C (per 1,000 PYs), the prevalence is presented as both certain (lower line of the shaded area) and certain + uncertain cases (upper line of the shaded area).

The prevalence of viral hepatitis B and C are displayed in Figure 2 and Supplementary Table 2. For HBV, the prevalence declined from 5.21 cases/1,000 PYs (4.16 certain cases/1,000 PYs) in 2013 to 2.99 cases/1,000 PYs (2.42 certain cases/1,000 PYs) in 2019 (−43%). For HCV, the prevalence declined from 1.50 cases/1,000 PYs (1.31 certain cases/1,000 PYs) in 2013 to 0.70 cases/1,000 PYs (0.55 certain cases/1,000 PYs) in 2019 (−53%).

The incidence rates are displayed in Figure 2B and Supplementary Table 3. In 2013, the incidence was 0.34/1,000 PYs for HBV and 0.25/1,000 PY for HCV. In 2019, the incidence was 0.12/1,000 PYs for HBV and 0.03/1,000 PY for HCV.

### Management of viral hepatitis in primary care

#### Hepatitis B patients

Among the 588 certain hepatitis B cases, 406 patients chronic hepatitis B patients were studied to assess the management of viral hepatitis in primary care. Among those, 289 patients (71.2%) were referred to a medical specialist at least once (Table 2). However, when studying the actual management, medical specialist performed HBV surveillance in 185 patients (45.6%) and the GP in 59 patients (14.5%). In total, 147 patients (36.2%) received no surveillance of their hepatitis B infection.

**Table 2.** Management of patients with viral hepatitis in general practice

	Hepatitis B *N* = 406	Hepatitis C *N* = 153	Coinfection *N* = 6
Referral hepatitis center, *N* (%)	289 (71.2)	136 (88.9)	6 (100.0)
Surveillance by GP, *N* (%)^a^		N/A	
Yes	59 (14.5)		0 (0)
No	147 (36.2)		3 (50.0)
N/A, surveillance specialist	185 (45.6)		3 (50.0)
N/A, cured infection	15 (3.7)		0 (0)
Ultrasound performed in patients without referral, *N* (%)	33/112 (29.1)	2/16 (12.5)	—
Medication, *N* (%)			
Yes	130 (32.0)	113 (73.9)	4 (66.7)
No indication for antivirals	94 (23.2)	1 (0.7)	0 (0)
No, other	174 (42.9)	33 (21.6)	2 (33.3)
Unknown	8 (2.0)	6 (3.9)	0 (0)
Curation, *N* (%)			
Viral suppression^b^	126 (31.0)	—	1 (16.7)
HBsAg^c^ loss	22 (5.4)	—	0 (0)
SVR^d^	—	82 (53.6)	2 (33.3)
Spontaneous clearance	—	3 (2.0)	0 (0)
No/unknown	258 (63.5)	68 (44.4)	3 (50.0)

Among certain chronic hepatitis B or C patients with at least 2 years of follow-up.

Surveillance was categorized as surveillance by the GP (at least 2 ALT checks within 3 years), no surveillance by the GP or medical specialist, surveillance by medical specialist (at least 1 letter or note of medical specialist about viral hepatitis B), or absent surveillance due to a cured infection; GP, general practitioner. ^b^Viral suppression with or without antivirals. ^c^HBsAg, hepatitis B surface antigen. ^d^SVR, sustained virological response.

To gain insight in the adherence of the updated GP guideline, we extracted a subcohort of patients, including certain cases of chronic hepatitis B patients who had at least 2 years of follow-up, including the period after March 2016 (when the GP guideline update was published), and who did not had HBV surveillance by medical specialist at that moment. In total, this subcohort included 226 patients of whom 148 patients (65.5%) received ALT surveillance at least once. Among these 148 patients, the mean number of ALT tests was 2.3 per patient (range 1–8) in 4 years. In addition, ALT levels were elevated (>35/45 U/mL for female/male) at least once in 34/148 patients (23.0%; range 36–275 U/mL). Consequently, after reviewing the medical records of those 34 patients with elevated ALT levels, 20 patients (58.8%) were referred to a medical specialist. Thus, no consequence was given to abnormal liver test in 14/34 patients (41.2%; mean 60 U/mL, range ALT 38–120 U/mL). Among those 14 patients, ALT levels were repeatedly increased in 8 patients (57.1%). HBsAg was only tested among 11 patients (4.9%).

#### Hepatitis C

Among the 153 certain hepatitis C cases who had at least 2 years of follow-up, 136 patients (88.9%) were referred to a specialized hepatitis treatment center (Table 2). In total, 113 patients (73.9%) received antiviral treatment; of whom 82 patients (53.6%) achieved SVR and 3 patients (2.0%) had a spontaneous clearance of the virus. Thus, 68 patients (44.4%) might still have a chronic hepatitis C infection on 2019 Dec 31, besides the 17 patients without referral to a hepatitis treatment center.

Tables 3 and 4 display the patient characteristics of, respectively, the 147 chronic hepatitis B patients and 68 chronic hepatitis C patients without adequate surveillance or successful treatment. Notably, among the hepatitis C patients, 54.5% had a registration of (prior) alcohol abuse (*P* = 0.005).

**Table 3.** Patient characteristics of chronic hepatitis B patients with inadequate management

	Hepatitis B Adequate management *N* = 259	Hepatitis B Inadequate management *N* = 147	*P*-value^d^
Sex (male), *N* (%)	147 (56.8)	68 (46.3)	0.042
Age at 31 December 2019, mean ± SD	50 (±14)	48 (±13)	0.153
Alcohol use, *N* (%)^a^			0.521
Never/socially active	121/136 (89.0)	57/62 (91.9)	
Alcohol abuses (prior or active)	15/136 (11.0)	5/62 (8.1)	
Body mass index (kg/m^2^), mean ± SD^b^	26.6 (±5.4)	27.4 (±4.4)	0.364
Liver-related comorbidities, *N* (%)			
Compensated cirrhosis	13 (5.0)	3 (2.0)	0.138
Decompensated cirrhosis^c^	4 (1.5)	1 (0.7)	0.448
Liver transplantation	0 (0)	0 (0)	0.451
Hepatocellular carcinoma	7 (2.7)	3 (2.0)	0.679

Alcohol use was stratified as none (defined as zero units/day or key words such as “alcohol”) or social use (defined as < 5 units/day or text words that indicated nonexcessive alcohol use), and alcohol abuses (defined as ≥ 5 units/day or ICPC code P15—chronic alcohol abuses). ^b^Body mass index measurements were available among 120 patients with adequate management and 58 patients with inadequate management. ^c^Decompensated cirrhosis was defined as ascites, esophageal or fundus varices, jaundice, and/or hepatic encephalopathy. ^d^Chi-square test.

**Table 4.** Patient characteristics of chronic hepatitis C patients with inadequate management

	Hepatitis C Not cured *N* = 68	Hepatitis C Cured *N* = 85	*P*-value^d^
Sex (male), *N* (%)	51 (75.0)	54 (63.5)	0.129
Age at 31 December 2019, mean ± SD	57 (±11)	57 (±9)	0.881
Alcohol use, *N* (%)^a^			0.005
Never/socially active	20/44 (45.5)	45/62 (72.6)	
Alcohol abuses (prior or active)	24/44 (54.5)	17/62 (27.4)	
Body mass index (kg/m^2^), mean ± SD^b^	25.1 (±5.1)	26.6 (±4.8)	0.196
Liver-related comorbidities, *N* (%)			
Compensated cirrhosis	9 (13.2)	12 (14.1)	0.875
Decompensated cirrhosis^c^	3 (4.4)	5 (5.9)	0.685
Liver transplantation	1 (1.5)	0 (0)	0.262
Hepatocellular carcinoma	2 (2.9)	1 (1.2)	0.434

Alcohol use was stratified as none (defined as zero units/day or key words such as “alcohol”) or social use (defined as < 5 units/day or text words that indicated nonexcessive alcohol use), and alcohol abuses (defined as ≥ 5 units/day or ICPC code P15—chronic alcohol abuses). ^b^Body mass index measurements were available among 54 patients with adequate management and 27 patients with inadequate management. ^c^Decompensated cirrhosis was defined as ascites, esophageal or fundus varices, jaundice, and/or hepatic encephalopathy. ^d^Chi-square test.

## Discussion

The Netherlands is a low-endemic country for viral hepatitis B and C (4,27). However, due to migration, low-endemic countries have local regions with high hepatitis endemicity, such as large cities as Rotterdam. Therefore, more insight in the prevalence and surveillance of viral hepatitis B and C in these areas is important. Using a large longitudinal observational dynamic database containing medical records of GPs in the area of Rotterdam, this study showed a decreasing prevalence of viral hepatitis B and C in primary care between 2013 and 2019. In addition, we demonstrated that the majority of patients with viral hepatitis B and C are referred at least once to a medical specialist. However, we found that a substantial part of the patients did not receive adequate surveillance or curative treatment.

In 2016, the WHO has implemented a global viral hepatitis elimination target (13). In this report, elimination was defined as a 90% reduction in new infections (95% for HBV and 80% for HCV) and 65% reduction in mortality by 2030, compared with incidence and mortality numbers of 2015. The interim targets for 2020 however, included a 30% reduction in incidence of viral hepatitis B and C in primary care. Our data showed a reduction of HBV incidence of 48% and a reduction of HCV incidence of 77% in 2019. This means that the Netherlands seems on track to reach the incidence target of the hepatitis elimination goal, in contrast to the results of a recent report (28). A decline in incidence has also been observed among other low-endemic countries such as United Kingdom and Iceland, possibly due to increased antiviral treatments using nationwide retrieval of lost to follow-up patients, and people who are imprisoned or inject(ed) drugs (29–31). However, the observed decline in incidence might also be caused by a potential decrease in diagnostic test for viral hepatitis in primary care, due to the barriers that GPs experience in case finding such as limited knowledge about viral hepatitis and subsequent risk groups or less attention for follow-up of abnormal liver tests (17,32). The increase of the number of GP practices in RPCD that originate from low-endemic areas of Rotterdam might also (partly) explain the decline in prevalence.

In the Netherlands, risk groups account for most cases of hepatitis B and C, including (first-generation) migrants, PWID, and men who have sex with men (MSM) (33–37). A possible explanation for the observed decline in prevalence could be the improved treatment options, especially for hepatitis C which can now eradicated with an 8- to 12-week cure with DAAs (38). However, an absent steep decline in prevalence after introduction of DAAs in 2015 among HCV patients indicates that other factors are also responsible for the decline in HCV prevalence, such as the improved harm reduction strategies for PWID and MSM, HBV surveillance among pregnant women and vaccination among children born from HBV-infected women.

In addition, we demonstrated that the referral rate to a hepatitis specialist was 71–89%. However, our data showed that 36.2% of the hepatitis B patients was not under surveillance by a hepatitis specialist or GP. Furthermore, we observed that many patients received ALT performance at least once, but an annual ALT check was only performed in the minority of patients. This is in line with the study results of Hofman et al. (39). In this study, the researchers observed a low performance of annual ALT monitoring among chronic hepatitis B patients in the period 2008–2015. This implies that the updated Dutch GP guideline has not been implemented sufficiently in daily practice. Moreover, we observed that 44.4% of the hepatitis C patients has not been successfully treated (or the GP has not been updated by the hepatitis specialist about viral eradication). In the Netherlands, the nationwide project CELINE has been initiated to retrieve ever diagnosed HCV patients who are not treated because they have become lost to follow-up (LTFU) (40). Our data suggest that GPs should initiate retrieval of their LTFU patients.

A possible explanation for the suboptimal surveillance of hepatitis B patients by the GP might be the lack of an appointment scheduling system. Consequently, the responsibility for the biannual ALT check lies with the patient instead of the GP. Another explanation could be limited knowledge about the updated guideline or viral hepatitis in general. This could be the consequence of the small number of viral hepatitis patients in every GP practice due to the low prevalence of viral hepatitis in the Netherlands (4). This has been confirmed in a recent Dutch qualitative study among GPs about case finding of hepatitis B and C patients showed that many GPs indicated that they have limited knowledge about the (updated) GP guideline, lack of time during a consult to address hepatitis screening and an insufficient registration system (32). Although this study reported barriers for case finding, we believe this barriers (and possible interventions) are also applicable for the suboptimal management for viral hepatitis. However, this warrants confirmation in another (quantitative) study.

Multiple interventions are needed to improve hepatitis B surveillance and retrieval of untreated HCV patients in primary care. First, an appointment scheduling system is warranted to invite hepatitis B patients biannually for their laboratory check. Second, IT changes, such as pop-up messages, could facilitate GPs to perform adequate HBV surveillance, referral of untreated HCV patients or screening among high-risk individuals. Hence, a registration system is crucial, including information about medical diagnosis, laboratory results, and background information such as country of birth. Third, GPs should be encouraged to participate in already available training courses for viral hepatitis. Fourth, a standard set of serum hepatitis markers and liver tests on laboratory forms could facilitate screening, which has been supported by the study of Helsper et al. (41). Finally, as mentioned above, retrieval of LTFU HCV and HBV patients can be worthwhile and should therefore be performed in every GP practice.

Despite that this is a large GP-based database, the following limitations need mentioning. Since migrants account for the majority of prevalent HBV and HCV cases, our results cannot be not directly translated to other (low-endemic) areas of the Netherlands, as our results are based on a multiethnic area in the Netherlands. Another limitation that should be acknowledged is the retrospective design and the fact that our results depend on the available data within the GP database, which is subjected to the input of individual GPs. Therefore, management and laboratory results of medical specialist are only available if the specialist sends communication to the GP. This could give an underestimation of the real number of patients that receive adequate HBV screening in the hospital or successfully treated hepatitis C patients. Since our data indicated that a few patients with liver cirrhosis or liver transplantation would not receive surveillance by a hepatitis specialist supports the suggestion that our data is limited by the retrospective design. Finally, due to privacy restrictions, if a patient changes GP within the network of affiliated GP practices of the RPCD, the patient enters the database with a new patient number. This could have resulted in duplicate cases. However, in the Netherlands, very few patients change of GP over time, when they do it is because of moving to a different region. Thus, the impact of duplicate cases to our findings is limited. In addition, for our calculations of the incidence and prevalence, we took the medical history into account. Therefore, a change of GP will not influence the incidence and prevalence rates.

In conclusion, we observe that the prevalence of viral hepatitis B and C is declining in a multiethnic area of the Netherlands. This implies that the Netherlands seems on track to achieve the WHO elimination target. However, many patients with hepatitis B and C might not receive adequate surveillance or antiviral therapy. It is therefore crucial to involve primary care in the road to complete elimination.

## Supplementary Material

cmac070_suppl_Supplementary_Tables

cmac070_suppl_Supplementary_Checklist

## Data Availability

Data not publically available.
