# Supplementary material for: Epidemiology and management of hepatitis B and C in primary care in the Netherlands: data from the Rijnmond Primary Care database
Source: Fam Pract. 2022 Jul 23;40(1):83–90. doi: 10.1093/fampra/cmac070 (PMC9909667; doi:10.1093/fampra/cmac070)
Supplement: cmac070_suppl_Supplementary_Tables [file cmac070_suppl_supplementary_tables.docx]

**Supplementary Tables**

**Supplementary Table 1. Follow-up**

|  | **Overall** | **Hepatitis B**  **N=717** | **Hepatitis C**  **N=252** | **Co-infection**  **N=8** |
| --- | --- | --- | --- | --- |
| **Mean follow-up period (months)**  No available time  <1 year  1-2 years  >2 years | 55 (19-98)  75 (7.7%)  111 (11.4%)  96 (9.8%)  695 (71.1%) | 57 (19-103)  55 (7.7%)  85 (11.9%)  70 (9.8%)  507 (70.7%) | 54 (21-96)  19 (7.5%)  26 (10.3%)  26 (10.3%)  181 (71.8%) | 54 (35-101)  1 (12.5%)  -  -  7 (87.5%) |

**Supplementary Table 2. Prevalence of viral hepatitis B and C**

|  | **Total PYs** | **Number of HBV cases** | **Number of HCV cases** | **HBV/1,000 PYs** | **HCV/1,000 PY** |
| --- | --- | --- | --- | --- | --- |
| **2013** | 101,860.04 | 424 - 531 | 133 - 153 | 4.16 – 5.21 | 1.31 – 1.50 |
| **2014** | 115,120.43 | 429 - 530 | 138 - 160 | 3.72 – 4.60 | 1.20 – 1.39 |
| **2015** | 119,483.61 | 426 - 531 | 135 - 160 | 3.57 – 4.44 | 1.13 – 1.34 |
| **2016** | 133,048.47 | 431 - 535 | 136 - 159 | 3.24 – 4.02 | 1.02 – 1.20 |
| **2017** | 146,935.38 | 435 - 537 | 120 - 143 | 2.96 – 3.65 | 0.82 – 0.97 |
| **2018** | 157,176.59 | 427 - 526 | 101 - 126 | 2.72 – 3.35 | 0.64 – 0.80 |
| **2019** | 162,214.48 | 392 - 485 | 90 - 114 | 2.42 – 2.99 | 0.55 – 0.70 |

Range of number of HBV/HCV cases, with corresponding prevalence per 1,000 PYs, was displayed as total certain cases – certain plus uncertain cases.

*Abbreviations: PYs, person years; HBV, hepatitis B virus; HCV, hepatitis C virus*

**Supplementary Table 3. Incidence of viral hepatitis B and C**

|  | **Total PYs** | **Number of HBV cases** | **Number of HCV cases** | **HBV/1,000 PYs** | **HCV/1,000 PY** |
| --- | --- | --- | --- | --- | --- |
| **2013** | 101,860.04 | 26 – 35 | 20 – 25 | 0.26 – 0.34 | 0.20 – 0.25 |
| **2014** | 115,120.43 | 26 – 26 | 18 – 23 | 0.23 – 0.23 | 0.16 – 0.20 |
| **2015** | 119,483.61 | 22 – 27 | 10 – 15 | 0.18 – 0.23 | 0.08 – 0.13 |
| **2016** | 133,048.47 | 23 – 24 | 9 – 10 | 0.17 – 0.18 | 0.07 – 0.08 |
| **2017** | 146,935.38 | 22 – 25 | 8 – 9 | 0.15 – 0.17 | 0.05 – 0.06 |
| **2018** | 157,176.59 | 13 – 15 | 7 – 9 | 0.08 – 0.10 | 0.04 – 0.06 |
| **2019** | 162,214.48 | 18 – 19 | 5 – 6 | 0.11 – 0.12 | 0.04 – 0.03 |

Range of number of HBV/HCV cases, with corresponding prevalence per 1,000 PYs, displayed as total certain cases – certain plus uncertain cases.

*Abbreviations: PYs, person years; HBV, hepatitis B virus; HCV, hepatitis C virus*
